# Lac Caninum

**Published:** 1884-04

**Authors:** E. W. Berridge

**Affiliations:** London


### LAC CANINUM.

E. W. Berridge, M. D., London.

In The Homœopathic Physician, vol. III, page 218, I published a cure by Lac caninum. This was dated December 28th, 1882. On April 23d, 1883, the same patient wrote as follows: "You have been so thoroughly successful in curing the nervous symptoms I complained of in my last letter, that I venture to ask if you will now kindly try to find a remedy for the sick headaches I am constantly troubled with. I have been suffering from them almost without intermission for the last three weeks. They begin at the nape, and gradually the pain settles in the right or left side of forehead. I have frequent attacks of severe vomiting; and when this is not the case, I have always a feeling of nausea and a fear to eat. On the 19th of this month, after vomiting all day, I had a rather severe faint in the evening, and have felt weak and out of sorts ever since. I have several boils on my left side; also two very angry gatherings, one under the nose, left side, and one up the left nostril; both came to a head, and discharged matter and blood, and afterward scabbed over; before the discharge there was shooting pain there; have also been troubled for the last month with paroxysmal gnawing pain in left upper canine tooth, which yields for a time to any cold application, but declines to take its final departure."

I was not surprised at the return of the sick headache, as she had had them at times for many years. I sent her six doses of *Lac caninum*$^{cm}$ (Fincke), one to be taken morning and evening. She received them on morning of April 24th.

On May 25th, 1883, she wrote: "When I tell you that for some weeks I have had a young lady in the house dangerously ill with congestion of the lungs and liver, and needing my constant attention, I am sure you will forgive my seeming neglect in not answering your last letter. The medicine had a wonderful effect; I have only had one sick headache since taking it, and that was brought on, I am sure, by over-work and worry. The first few doses affected the eyes and made me feel as if the eyeballs were too large and I must move them about. The breathing was also difficult; I could not get a full breath."

On August 9th, 1883, she wrote: "I have not had one of my bad headaches since taking your last remedy. Once or twice when I have had food not quite suitable to the stomach, my head has ached slightly, but the pain has never lasted or become bad as formerly. All the other symptoms disappeared, so we may presume the medicine was correct and suitable in every way."

